# Integrating machine-readable user interface requirements into open networked operating rooms

**DOI:** 10.3389/fdgth.2025.1520584

**Published:** 2025-07-21

**Authors:** Okan Yilmaz, Dominik Stegemann, Klaus Radermacher, Miriam Lange, Armin Janß

**Affiliations:** ^1^ Chair of Medical Engineering, Helmholtz-Institute for Biomedical Engineering, RWTH Aachen University, Aachen, Germany; ^2^ SurgiTAIX AG, Herzogenrath, Germany

**Keywords:** usability engineering, UI Profile, user interface profile, user interface requirements, IEEE 11073, basePKP, participant key purpose, SDC

## Abstract

Comprehensive risk management (**RM**) and usability engineering (**UE**) must be performed to enable safe and usable interoperable medical device systems (according to IEEE 11073 SDC). This has to be fulfilled by applying recognized standards such as ISO 14971 (RM) and IEC 62366-1 (UE). Addressing the complexity of use cases with multiple network participants requires defining use context, hazardous situations, user profiles, user interfaces, system function contributions, limitations, configurations, and required conditions for safe use. We propose extending the categories mentioned in IEEE 11073-10700 with standardized user interface requirements provided by medical device manufacturers. A consumer of networked services can consider those UI Profiles containing design-, risk-, and process-related UI requirements during its design phase, usability engineering process, and risk management. This allows a systematic deficiency analysis prior to device usage, encompassing human-induced risks and thereby enhancing usability, patient safety, and finally operational efficiency. Using benchmarked, verified, and tested UI controls to create user interfaces that fulfill those requirements automatically might also be a solution for the future. This work presents an architectural overview incorporating ISO IEEE 11073-10700 standard requirements. Significantly, it extends these standards by introducing categories that enhance support for the usability engineering and risk management process, emphasizing the role of UI Profiles in achieving safe and usable operating room environments with more flexibility regarding interoperability and enabling a human-induced risk analysis prior to device usage. The number of surveyed manufacturers (8) and the need for real-world validation are limitations of this work, which should be validated by future work.

## 1 Introduction

A medical device’s user interface must be designed according to previously specified User Interface (UI) requirements, verified, and validated during the conformity assessment. The situation becomes more complex when open interoperability, according to the IEEE 11073 SDC (**S**ervice-oriented **D**evice **C**onnectivity) standard family, is introduced within the operating room (**OR**) or the clinic. SDC defines a communication protocol that enables medical devices to communicate with each other over the network by providing technical device descriptions and functionalities. In open networks, multiple types of participants are involved, where one provides device functionalities (**SDC Provider**), and the other consumes/uses such services (**SDC Consumer**). Typical use cases are remote data display or remote device control. Usability-related errors remain a leading contributor to adverse events in healthcare (1–3).

The technical documentation becomes a challenge if the SDC Consumer doesn’t have enough information from the SDC Provider. Providing private, sensitive information to third parties is not in the interest of the SDC Provider since it contains internally valuable and critical information. Nevertheless, the SDC Consumer has to develop some kind of user interface based on the provided information and their own understanding of the device. The SDC Provider has a very deep insight into its own device. Performing risk assessments and applying and providing UI Profiles can substantially harness the expertise of the Provider and mitigate potential risks for the Consumer. With this method, foreseeable risks can already be considered during the design phase and be tested against in the verification phase. This information could be provided using different methods, but so far, no standardized, machine-readable language exists to do this.

Both SDC and UI Profile usage can significantly impact patient safety and clinical workflow in the operating room. While interoperable medical device systems create use-cases, such as remote control or remote monitoring, they also introduce additional risks: Inconsistent user interfaces, mismatch between remotely displayed and device information (e.g., different units), unintuitive interfaces, or just missing essential information for safe device operation. The controls used for critical tasks need to be designed in a way to prevent accidental activation, for instance, by requiring the user to confirm critical adjustments (1) or implementing design modifications (2).

These risks can interrupt clinical workflows, increase a user’s mental workload, and overall increase the risk of human error. By embedding UI Profiles into the development and evaluation process, foreseeable device-related risks can be identified and mitigated, thereby increasing patient safety and improving clinical workflows.

Finally, a Health Delivery Organization needs testing tools and processes to operate such interoperable systems. Without those, having open SDC interoperability will still be limited to manufacturer-to-manufacturer collaborations and solutions.

## 2 SDC—service-oriented device connectivity

The BMBF (German Federal Ministry of Education and Research) lighthouse research project “**OR.NET**—Secure Dynamic Networking in the Operating Room and Clinic” (2012–2016, funding no. 16KT1238) aimed to develop the technical basis for safe and dynamic networking of components in the operating room and clinic. An architecture and communication language were developed, and novel approaches for trust and distribution of responsibilities in open networked systems. The SDC Standard family consists of three parts, which will be explained in detail in the following subchapters (4, 5).

### 2.1 SDC core standards

The SDC core standards make technological interoperability possible. By providing a foundation, structure, and semantics, devices can communicate, discover each other, and interpret messages in a standardized way.

**ISO/IEEE 11073-20702:** “Medical Devices Communication Profile for Web Services,” also known as MDPWS, enables the foundation for interoperability by providing the ability to exchange data safely and discover network participants in a distributed network via web services. It can be viewed as an extension of **DPWS** for medical purposes (6).

**ISO/IEEE 11073-10207:** “Domain Information and Service Model for Service-Oriented Point-of-Care Medical Device Communication,” also known as **BICEPS** (Basic Integrated Clinical Environment Protocol Specification), provides a semantic description of medical device capabilities and state information using a participant model (**MDIB**) and communication/message model. It establishes a common language and structure to exchange health-related information (7).

**ISO/IEEE 11073-10101:** Nomenclature and other coding systems define how medical information is coded and categorized. This helps different healthcare devices and systems understand and exchange information accurately. This nomenclature supports both the domain information model and service model components and the semantic content exchanged with medical devices (8).

**ISO/IEEE 11073-20701:** “Point-of-care medical device communication-Service oriented medical device exchange architecture and protocol binding” **defines the service-oriented architecture and specifies bindings** toward other standards such as NTP, Differentiated Services, QoS Requirements, −20702, and −10207. Due to its binding nature, it is often referred to as “SDC Glue.” (9).

### 2.2 Safety, trust & participants’ responsibilities

The Participant Key Purpose (PKP) is a set of requirements that support manufacturers in making valid assumptions about other network participants. This allows them to perform risk management, verification, and usability engineering for the safe use of device functions. It also specifies requirements for the allocation of responsibilities. It is split into four parts, two of which are in the proposal state and two of which are already finished standards.

**IEEE 11073-10700:** “Standard for Base Requirements for Participants in a SDC System” specifies requirements for allocating **responsibilities** to SDC participants. Those enable manufacturers to perform risk management, usability engineering, as well as verification and validation (10).

**IEEE 11073-10701: “**Metric Provisioning by Participants in a SDC System” **defines requirements** for SDC participants to enable safe and secure contribution to clinical functions based on the **exchange of metric information**; this includes remote display, partial automation of diagnosis and therapy, and changing settings based on received metric information (11).

**IEEE P11073-10702:** “Standard for Alert Provisioning by Participants in a SDC System” defines requirements to “[…] to exchange alarm data and related remote control in a manner that improves safe, secure and effective contribution to the functionality of a distributed system.” (12).

**IEEE P11073-10703:** “Standard for External Control by Participants in a SDC System,” will define the rules, methods, and requirements for external control.

### 2.3 Device specializations

**IEEE P11073-10720:** “Module Specifications for a Service-Oriented Medical Device Exchange Architecture” specifies how to represent device components in a network, defines the device-type independent use of term codes, and outlines communication rules, while it doesn't provide detailed rules for specific devices (13).

The **11073-1072X** standards define the scope, structure, and semantics of information and functionalities offered by a specific class of devices. These encompass parameters like dependencies, remote control commands, technical device descriptions, behavior in various states, and networking requirements. The standards provide a framework to adhere to, enabling devices to assume roles in networked systems (14). The research project **PoCSpec** (Modular Specialisations for Point-of-Care Medical Devices) developed standards for devices in the field of high-frequency surgery and endoscopy. By conducting regular meetings with those vendors, consent was found between the manufacturers for a standardized device standard (15).

## 3 User interface profile

The device specializations (IEEE 11073-107XX) describe how medical devices present themselves (their available functions) in an SDC network and the technical requirements other network participants must comply with to interact with them in a safe way. So far, those device profiles do not include HMI characteristics, which are necessary for remote display and device control and especially for safe and usable interfaces. Previous work identified and addressed a need for user interface requirements (16–22).

A device-specific user interface profile was proposed, and different but very similar definitions exist to date:

• 2016 from Thorn et al. (23): The UI profile describes devices’ requirements for displaying their features and functions on other devices. These are guidelines for visualizing metrics and triggering actions based on information from ISO 24752 (Universal Remote Console). However, it does not prescribe the specific design of the user interface but provides limitations and suggestions for designing the interface.
• 2018 from Janß et al. (20): “Characteristics of input and output devices as well as GUI interaction elements (size, position, etc.) and their dependencies, criticalities of functions, grouping and positioning information regarding interaction elements, etc., scenario-specific defined performance shaping factors (PSFs), e.g., environmental factors”
• 2022 from Yilmaz (author of this document) et al. (16): “A device-specific set of requirements and specifications regarding Human Machine Interactions a network subscriber must fulfill, in order to operate medical device functions or to display medical device properties.”

**Figure 1:**
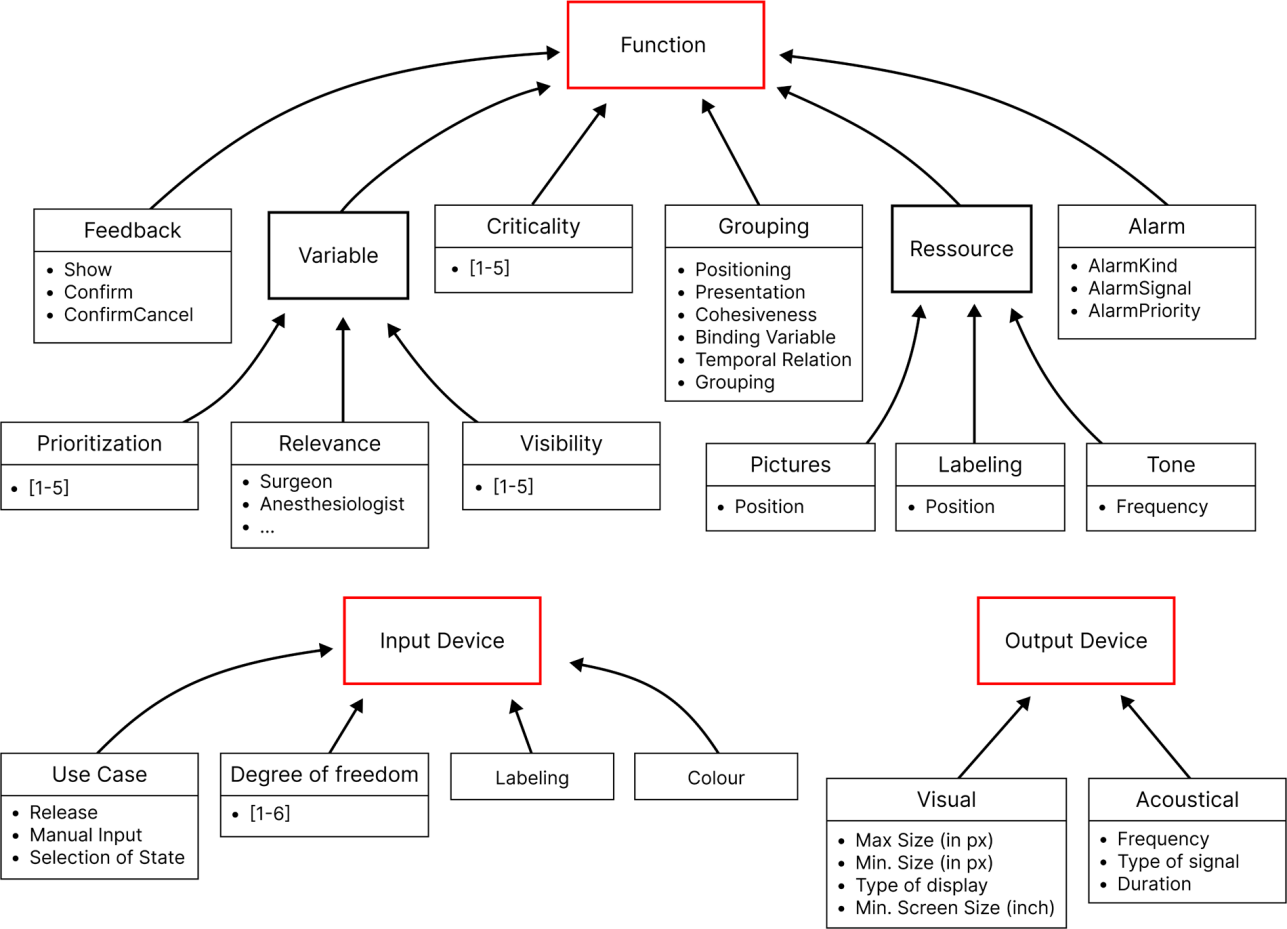
Previous UI profile emphasizing detailed grouping and dimension specifications, with stronger focus on rigid technical requirements.

In earlier versions, the User Interface Profile was based on ISO 24752, VDI 3850, Hölscher, Preim, Fellbaum, DIN EN ISO 7731, DIN 894-1, DIN 894-2, DIN EN 60073, and ISO 9241 family (such as −400, −420, −410, −303,). Figure 1 illustrates an earlier version of the UI Profile, outlining a more rigid categorization of interface elements and technical requirements. This legacy approach places strong emphasis on grouping, positioning, and specifying fixed dimensions and solutions

**Figure 2:**
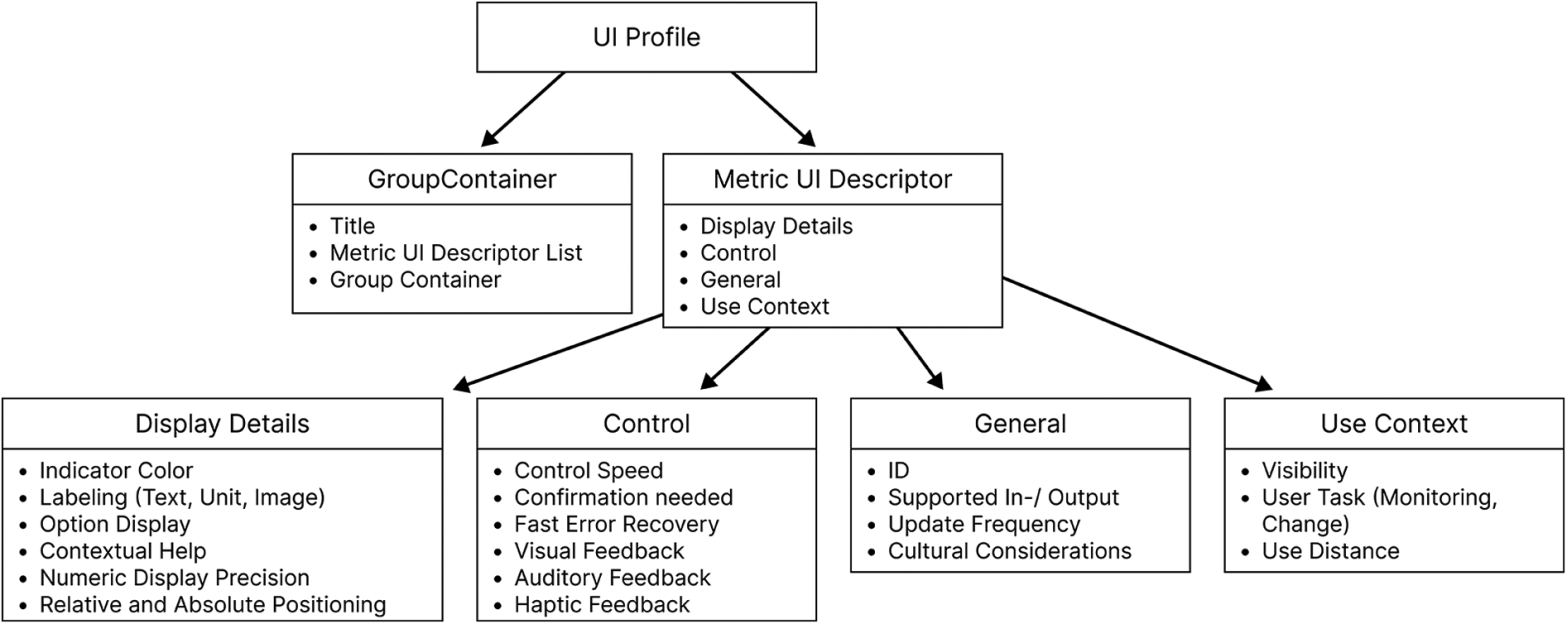
Current UI profile—an updated framework that integrates additional risk management attributes and user interaction requirements, offering greater flexibility.

The approach from Janß et al. focused heavily on grouping, positioning items, and the technical specifications of input and output devices. This increases the chances of developing usable interfaces but may limit the design freedom of a potential user. It also contains technical restraints and proposes concrete dimensions for visual elements. This version has been overhauled to develop the current version of the UI Profile (See Figure 2 and Table 1). It now doesn’t specify user interface controls or control mechanisms but focuses additionally further requirements directly derived from risk management and (e.g., SDC-) standards (control speed, feedback mechanism, user task, update frequency, labeling, display precision, etc.) in addition to the already considered usability engineering requirements (effectiveness, efficiency, feedback, labeling) (16). We used clinical use-cases to identify device-specific UI-requirements. The use-cases included tele-supervision (24), ventilator development (25), common neurosurgery (26), and dorsal cervical decompression and spinal fusion for myelopathy treatment employing surgical navigation. The categories of the UI Profiles were iteratively presented and discussed with medical device manufacturers, designers, and software developers. Requirements from particular device standards such as the IEC 60601-2-2 for high frequency devices were also included as far as applicable.

**Table 1:** User interface profile categories.

Title	Aspect	Description
Visibility	Perception	The visibility level describes at what time a UI control should be perceivable.
Control Speed	Perception, Cognition, Motoric	This describes the required time to change an option, whether numerical or non-numeric or to activate an option. The number of interaction steps correlates with the time it takes.
Option Display	Perception	This category describes whether the available options of a selection should always be fully visible (e.g., Radio Buttons) or could be hidden and be shown after an interaction step (e.g., Dropdown)
Residual probability of error	Safety	This category addresses the chance of wrong perception, cognition, or motoric actions while interacting with a UI.
		Depending on the use case, this probability should be very low for critical use cases (drug overdose) or can be medium-high, where no harm is possible (changing screen brightness).
User task	User Model/ User Interaction	This category differentiates the purpose of the option/value/task.
Safety Classification	SDC	This classifies the Safety of a metric according to IEEE 11073-10207 (7)
Labeling	Cognition, Perception	This category describes if an UI Element needs an additional description next to its value
Feedback mechanisms	Perception, Cognition, Motoric	This category describes how and when visual, auditory, or haptic feedback should be given
Contextual Help	Cognition, Perception	This category describes whether help information should be accessible and what it should contain. Useful in rarely used or critical situations.
Grouping/Relative/Absolute Positioning	Perception	This category describes which metrics should be displayed together.

The goal was to systematically consider requirements from the different stakeholders involved. They were also applied several times by different users on medical devices (e.g., endoscopy, high-frequency device, OR-light, infusion pump, and OR-table), and some interfaces have already been evaluated by clinical users. The formative evaluation of a user interface developed based on using UI Profiles will be part of an upcoming publication. Usability evaluations of SDC Workstations have already taken place in smaller studies (24, 27, 28).

UI Profiles specifications can be included in an early design phase and used to design a user interface that meets specifications determined by an SDC Consumer. In addition to HMI-related risks, the technical specifications of input and output devices also play a vital role. Wickel et al. proposed technical attributes (idle state, actuation force threshold, actuation area ratio, manual precision, etc.) for input devices to be included in the ISO IEEE 11073-10207 (17).

## 4 Benchmarking of GUI elements

While developing a graphical user interface (GUI), a designer has to consider the user’s knowledge, expertise, goal, experience, environment, internal and external shaping factors, as well as their mental workload and other upcoming performing shaping factors.

In addition, a medical device’s GUI has to meet regulatory requirements regarding risk and usability engineering, including safety, efficiency, effectiveness, learnability, and user satisfaction, according to IEC 62366-1. The chosen UI elements heavily influence those categories. A radio button might be faster when selecting one of two options, while a dropdown might be faster for numerous options (29).

Almost all modern design guidelines have addressed the usage of GUI elements and their characteristics; examples are shown in (30–36). Those guidelines consider the number of options, the type of task, the input device, size, and additional task-related factors to create selection tables and matrices over the years, containing partly HMI-related criteria (1, 37–39).

In previous work, UI elements from those guidelines have been collected for mutually exclusive, non-mutually exclusive, and numeric selections (40). Those UI Elements have different efficiency, accuracy, and error rate characteristics while having different labels and sizes. Different studies determined some of those characteristics in 1995 (37) and 1999 (38). Since those studies were performed more than 25 years ago, a lot has changed. Tablets have higher resolutions, are more responsive, and allow for faster interaction. UI elements have simple animations during interactions, and their design has changed (e.g., rounded corners, bigger interaction fields, more compact and animated). More UI elements have been developed since then [e.g., animated toggle buttons (31), chips (33)], and some actions stemming from hardware buttons have been transferred to touchscreens (e.g., swipe, rotate, tap and hold).

Some studies use “expert knowledge” or see the rendering task as an optimization problem to determine the right UI elements, their positioning, and their grouping (41). A study to determine objective criteria to define UI elements’ efficiency, effectiveness, and error rate is currently being conducted and could, therefore, in the future, serve as a filter when choosing appropriate UI elements using the UI Profile.

## 5 SDC entities and their responsibilities

SDC Communication uses a service-oriented medical device architecture (SOMDA), which has been standardized as part of the IEEE 11073 SDC Standard (42). In this chapter, we will focus on tasks, responsibilities, and requirements of different participants in the SDC Architecture. The results of several research projects and manufacturers’ efforts have been written into the SDC standards. The PKP defines a set of requirements that support manufacturers in making valid assumptions about other network participants. This enables performing risk management, verification, validation, and, therefore, risk analysis and usability engineering for the safe use of device functions. It also specifies requirements for the allocation of responsibilities (10–12).

Several meetings were conducted with the IG-NB (alliance of notified bodies for medical devices in Germany), during which concepts for risk management in open networked solutions were presented and discussed. In the latest Gemini SDPi Ecosystem Pathway Summit 03/2024, a broad set of stakeholders, including the FDA, IG-NB, 13 medical device manufacturers, and two research institutes, discussed ongoing challenges in SDC, including the regulatory pathway for market approval (43).

**Figure 3:**
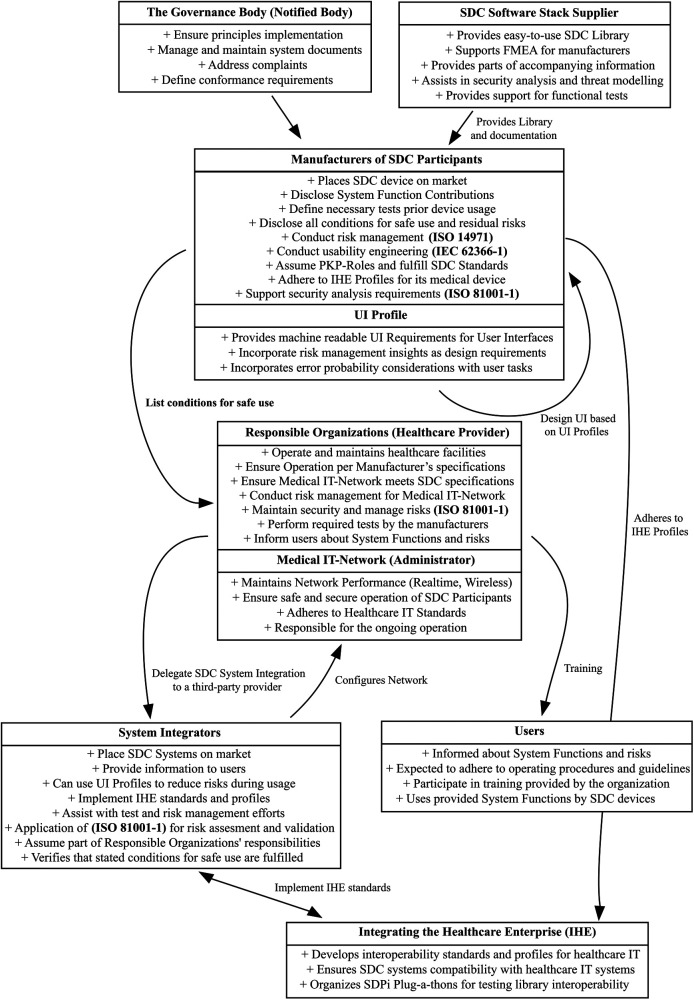
SDC actor graph—a simplified overview showing all stakeholders in the SDC ecosystem and how UI profiles could be integrated.

Figure 3 provides a high-level overview of SDC actors, their responsibilities, tasks, and where a UI Profile could be included. It shows their respective roles and their relations with each other. Directly affected participants in such a system are

• Responsible Organization (Also called Healthcare Delivery Organization)
• Medical IT Network (Often part of the Healthcare Delivery Organization)
• System Integrator [“Organizations that place SDC Systems on the market” (44])
• Medical Device Manufacturer
• Users (Including clinical and non-clinical staff)

Indirectly affected participants are

• SDC Software Stack Supplier
• The Governance Body (Notified Bodies)
• Integrating Healthcare Enterprise (IHE)

While Figure 3 looks overwhelming, it might be noted that it is an attempt to simplify 20 years of interoperability research into one simple model without leaving out any stakeholders.

### 5.1 Accompanying information

A medical device manufacturer has to provide information accompanying a medical device. The DIN EN ISO 20417 defines **Accompanying Information** as information for the medical device’s installation, use, decommissioning, and disposal (45). In SDC Systems, the accompanying information is necessary for outlining system functions, user requirements, conditions, and use context that must be met for system functions to be available. Such conditions can include functional tests, redundancy of resources and networks, and labeling requirements (10).

The instruction for use is part of the accompanying information. It contains user-directed information essential for the safe and effective use of a medical device or accessory (10, 45).

### 5.2 Risk management in SDC networks

Risk Management is still a challenge in open networked solutions. There is an ongoing working group in the OR.NET association (“Conformity assessment and regulatory requirements”), which coordinates with the interest group of notified bodies in Germany (IG-NB) to establish a strategy for the approval of open networked medical devices (46). We will give a short overview of challenges, the current approach, and ongoing work.

#### 5.2.1 Current challenges

When an SDC Provider provides its functionalities to SDC Consumers, the **Use Specification** of the SDC Consumer, first of all, is, in general, unknown to the SDC Provider. The SDC Provider is responsible for providing his functionalities in the way he documented them and by showing conformity to SDC PKP standards. An SDC Consumer performs Risk Management using this information and its Use Specification.

Use Specification is defined in IEC 62366-1 as a “summary of the important characteristics related to the context of the use of the medical device.” This includes the intended medical indication, patient population, intended part of the body to be interacted with, the intended user profile, the use environment, and the operating principle (47).

In addition, external performing shaping factors also influence device usage. Examples are situational characteristics (lighting, noise, temperature), task and equipment characteristics (nature of task, ergonomic, complex or poor interfaces), and job and task instructions (clear/unclear or poorly written instructions, comprehensive/missing training) (48). The setup of the operating room, frequency of use, and the positioning of the system might also influence the user when interacting with medical devices and need to be considered during risk management (47).

When using shared resources such as screens, network bandwidth, or input devices, a proper allocation is mandatory. This is part of the stated conditions for safe use and needs to be fulfilled. The system owner is responsible for verifying this and performing functional tests before device usage. Network issues such as insufficient bandwidth, loss of connection, malicious data (cybersecurity), wrong patient/location association, wrong device pairing, and use errors contribute to new causes and hazards and must be considered during a risk analysis (10).

Security-related issues need to be addressed by a shared threat modeling and analysis from device manufacturers, healthcare providers, and the library supplier, as mitigations to cybersecurity risk will fall under the responsibility of all involved parties. An IEC 62304 documented library, which analyzed potential threats, could support risk management (such as sdcX from SurgiTAIX). Another important challenge is creating, distributing, and managing SSL certificates for SDC devices. Currently, there are no established processes for manufacturer-independent certificate management. This will be addressed in the research project Medi.NET (2024–2027).

#### 5.2.2 Usability challenges

The Usability Engineering process and risk management of an SDC Consumer must ensure that the use-related risks are identified and mitigated, and that risk control measures are effective. This presupposes that all causes of hazards are identified in the risk analysis of the SDC Consumer.

While an SDC Provider can specify which requirements need to be fulfilled by an SDC Consumer, this list of requirements is, by all means, not complete because the SDC Provider does not know in which use context and from which SDC Consumer their device will be controlled. The responsibility to state an interoperable use specification (Use Environment, User Profile) lies with the SDC Consumer. It is the basis for risk management and usability engineering.

We propose that the SDC Provider add standardized, machine-readable user interface information to its device profile. This has already been proposed, but no machine-readable schema (e.g., XSD) has been defined in SDC yet (19, 20, 22).

#### 5.2.3 Feasibility of integration into existing regulatory frameworks

UI Profiles, as proposed in this paper, can become a method to systematically support regulatory requirements stated by the MDR and FDA. Manufacturers can integrate UI Profiles into their design dossier (supporting development, risk management, verification, and validation) to demonstrate that SDC Consumers have systematically considered the new use context and other network participants, as stated in the Base PKP Standard.

For instance, referencing UI Profiles through GUI development and evaluation could illustrate how following standardized labels and symbols, requirements for input controls, positioning, grouping, and feedback mechanisms meet other SDC Participants’ requirements for safe and usable interfaces.

While the approach will require further alignment, the UI Profile concept offers a structured format that could streamline regulatory submissions by clarifying how user interface design decisions address or mitigate relevant use errors.

## 6 Questionnaire: integrating user interface profiles into the SDC architecture

The primary goal of this chapter is to determine if UI Profiles are necessary, beneficial during the design and verification phase, capable of mitigating risks prior to device usage, and feasible for use in developmental stages.

To evaluate the effectiveness and practicality of UI Profiles, a questionnaire was filled out by eight medical device manufacturers who participated in the “SDPi Developer Workshop 2024” (49). The workshop was chosen since medical device manufacturers participated with experience in risk management, SDC interoperability, and usability engineering. The participation was voluntary. The questionnaire was designed to gather feedback in four key areas: the necessity of UI Profiles, their usefulness during the design process, their ability to preemptively address risks, and their feasibility for usage by the manufacturers. Pre-testing was performed with associated research assistants with experience in SDC, usability engineering, and risk management.

**Figure 4:**
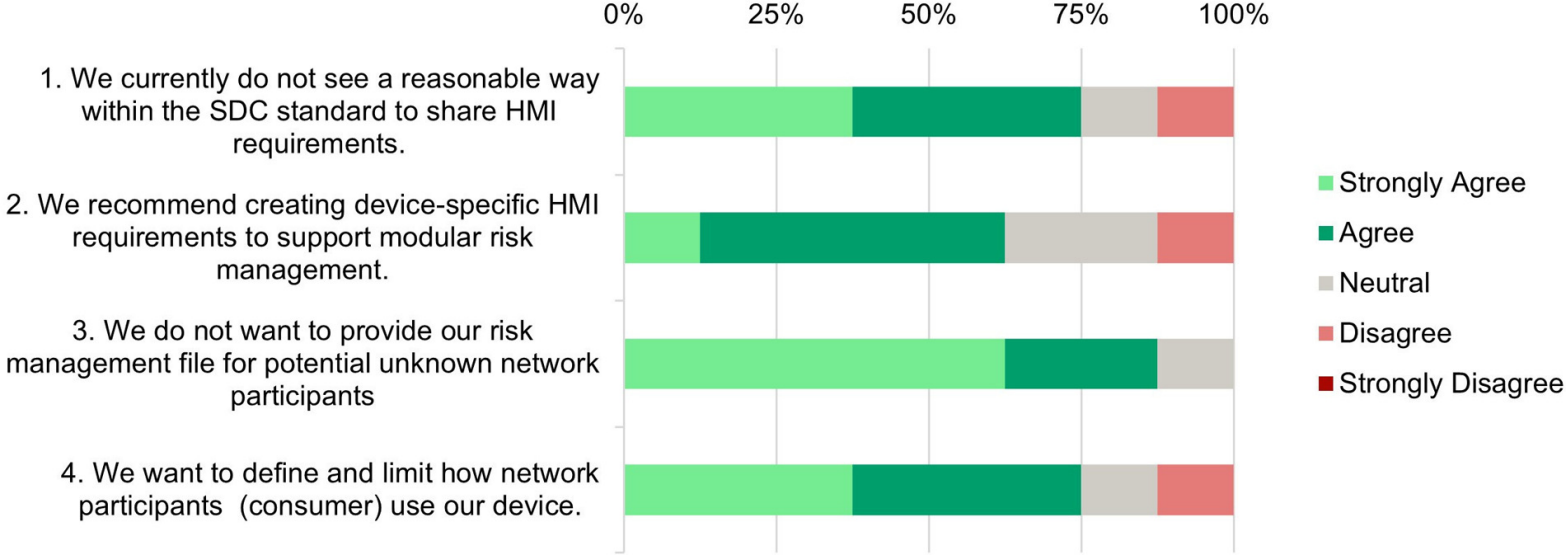
Need assessment—survey responses from medical device manufacturers about the necessity of standardized user interface profiles.

**Figure 5:**
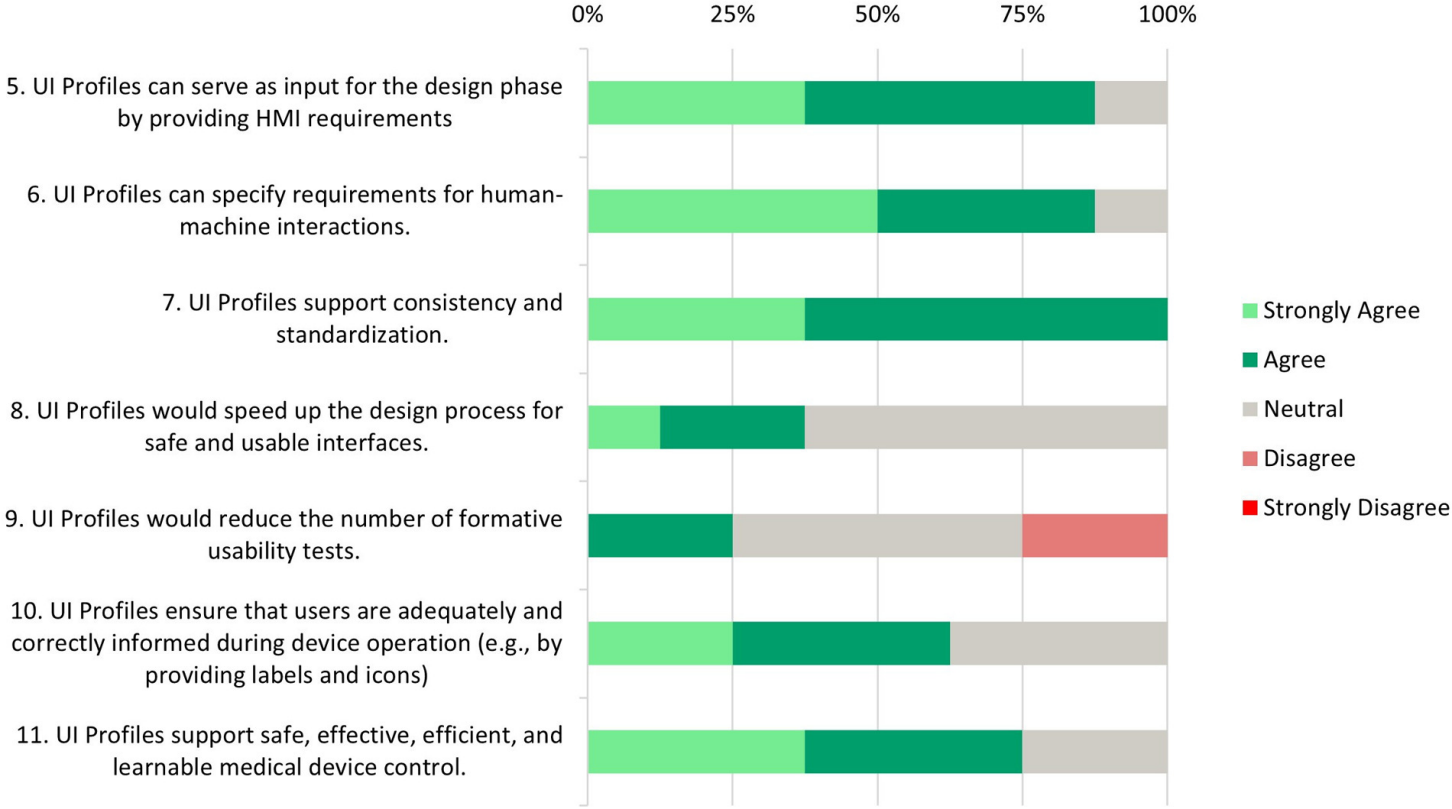
Development and testing phase—survey responses from medical device manufacturers about UI profiles support and needs during the design, development and testing phase.

**Figure 6:**
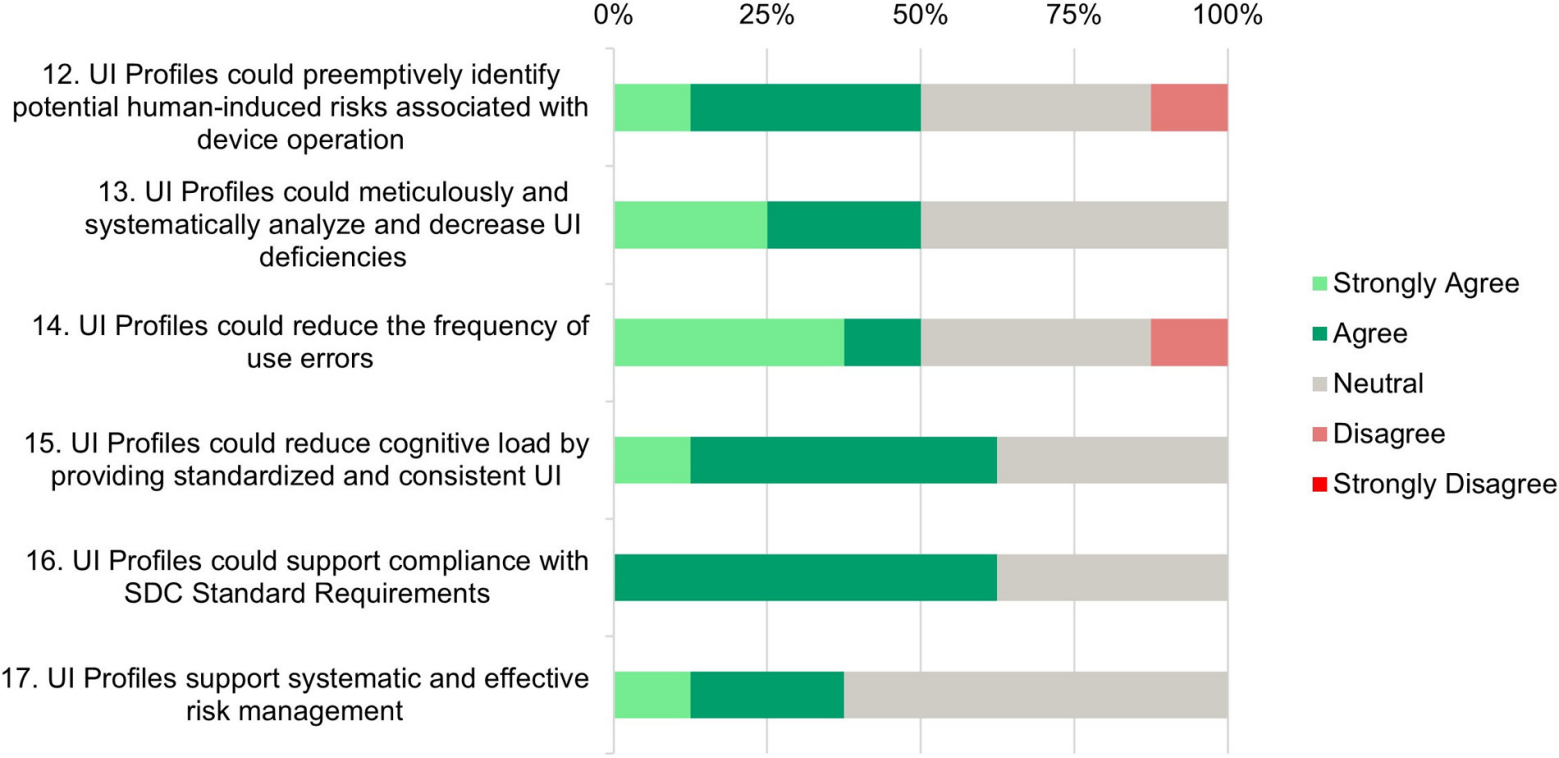
Risk analysis support—survey responses from medical device manufacturers about their perception in the areas of identifying or mitigate human-induced risks.

**Figure 7:**
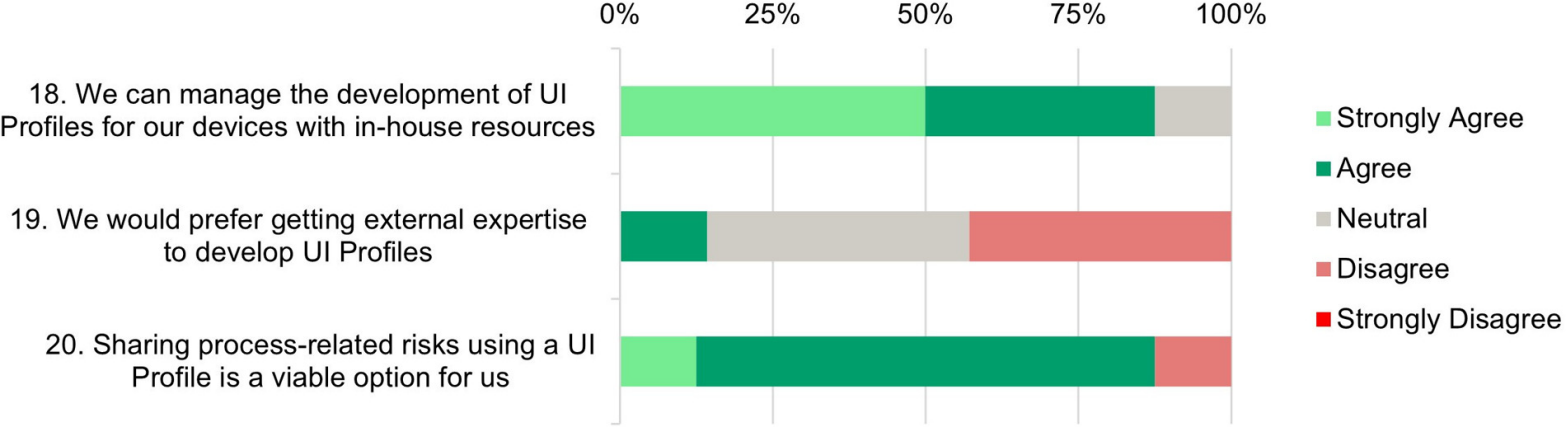
UI profile creation process—survey responses from medical device manufacturers about their ability and readiness to use UI profiles.

Each participant was asked to rate 20 statements on a Likert Scale from 1 (Strongly Disagree) to 5 (Strongly Agree). The results are displayed in Figure 4 **(Need Assessment)**, Figure 5 **(Development and Testing Phase)**, Figure 6 **(Risk Analysis Support)**, and Figure 7 **(UI Profile Creation Process)** and provide insight into manufacturers’ views toward UI Profiles.

### 6.1 Data analysis

The following subchapter discusses the rated statements in the questionnaire as part of a descriptive and statistical analysis. We performed a One-Sample Wilcoxon Test to determine if the participants’ answers are significantly tilted toward agreement or disagreement rather than remaining near the midpoint (Neutral). The results can be found in the attachment as well as behind every statement in the descriptive part (* for statistically significant) (50).

### 6.2 Need assessment

**Statement 1:** A significant number (75%) agree that the current SDC standard lacks effective methods to share HMI requirements. This highlights a gap in the SDC standards to ensure the interoperability and usability of open integrated systems through standardized HMI specifications.

**Statement 2:** Most respondents (62.5%) favor the creation of device-specific HMI requirements to support modular risk management. It indicates a need for developing more comprehensive guidelines within the SDC framework to facilitate sharing HMI requirements.

Statement 3*: There is a strong consensus among manufacturers (87.5%) not to share their own risk management files with unknown SDC network participants. This reflects concerns about the confidentiality of sensitive information. It underscores the need for safe and trusted mechanisms within the SDC framework to transfer risk-related requirements for safe device use.

**Statement 4:** Most respondents (62.5%) want to define and limit how their device is being used in an SDC network. That means that there is a need to transfer requirements about device usage to potential network partners.

### 6.3 Development and testing phase

Statements 5*, 6* and 7*: Most participants (87.5%) (strongly) agree that UI Profiles could serve as input for the design phase and that UI Profiles can specify those HMI requirements. All participants unanimously agree that UI profiles support consistency and standardization.

**Statements 8 and 9**: While some respondents (37.5%) (strongly) agree that UI profiles can accelerate the design process, a significant portion (62.5%) remains neutral. The idea that the number of formative tests would be reduced shows a mixed reaction, where 50% stay neutral, 25% agree, and 25% disagree.

**Statement 10:** Most participants (62.5%) agree that UI profiles are able to ensure that users receive adequate and correct information during device operation. A UI Profile delivered by a medical device manufacturer supports this by providing clear and standardized labels, units, and icons. However, 37.5% of neutral responses suggest that some participants may have reservations about the flexibility of UI profiles in addressing all information needed during device operation.

In a subsequent conversation, a manufacturer commented that flexibility is important for his medical device, where a confirmation window could be conditional. Certain numeric values might lead to unsafe/dangerous states when changing a property. This is influenced by multiple parameters, not just the metric itself. Multiple formulas are used, and requirements are checked to determine whether a confirmation window should appear. Currently, modeling such scenarios using the existing capabilities of the UI Profile or SDC is impossible. Whether such technical, calculated, or simulated information used for risk mitigation should be part of the MDIB or the UI Profile is debatable. This decision should be up to manufacturers working on a ventilator device specialization.

Statement 11*: A significant number of respondents (75%) (strongly) agree that UI profiles support safe, effective, efficient, and learnable control of medical devices. This suggests a strong belief in the value of UI profiles in improving medical devices’ overall quality and usability. 25% of neutral responses might indicate questions about their practical application in all areas.

### 6.4 Risk analysis support

**Statements 12 and 13:** While 50% of participants (strongly) agree that UI Profiles could preemptively identify human-induced risks, the other half either remains neutral (37.5%) or disagrees (12.5%). This suggests a need for more evidence regarding the effectiveness of risk identification.

Half of the participants (strongly) agree that UI Profiles can systematically analyze and reduce UI deficiencies, while the other half remains neutral. This neutrality reflects the identified need for more evidence.

**Statement 14:** A majority (50%) (strongly) agree that UI profiles could reduce the frequency of use errors, indicating a belief in their potential to improve user interaction and reduce errors. However, 37.5% of participants remain neutral, and 12.5% disagree. This further highlights the need to demonstrate how UI Profiles can effectively reduce errors.

**Statement 15:** The majority of respondents (62.5%) agree that UI profiles can reduce cognitive load through standardization, which is a positive indicator of their potential to enhance user experience and operational efficiency.

**Statement 16:** A majority (62.5%) agree that UI profiles can support compliance with SDC standard requirements, indicating confidence in UI Profiles’ ability to help meet regulatory and standardization needs. Currently, only a few SDC medical devices are on the market, and those are either from one manufacturer or B2B solutions. This allows comprehensive risk management on the basis of shared documents. Open modular risk management cannot yet be conducted because the SDC-PKP has not been finalized. The idea of providing requirements for safe use in a standardized way is not yet in the minds of manufacturers, but it will be essential in the future.

**Statement 17:** The majority of participants (62.5%) have expressed neutrality towards the idea that UI profiles support systematic and effective risk management, while 37.5% (strongly) agree with this statement. The lack of disagreement highlights that there is no opposition to the concept, indicating a generally positive attitude toward the potential benefits of UI Profiles in this area. The high neutrality suggests that practical examples are needed to illustrate how UI Profiles contribute to a more effective risk management process.

### 6.5 UI profile creation process

Statements 18* and 19: The last three statements focus on the process of UI Profile creation. 87.5% of the participants (strongly) agree that they could develop UI Profiles for their own devices with in-house resources. Only 12.5% would prefer to get external expertise to develop UI profiles. This emphasizes confidence in the manufacturers’ internal capabilities and in-house usability and risk management knowledge.

**Statement 20:** An overwhelming majority (87.5%) of participants (strongly) agree that sharing process-related risks using UI Profiles is a viable option for them. This is a positive outlook and suggests that there is value in leveraging UI profiles to facilitate transparent and effective sharing of risks among SDC stakeholders. This broad acceptance and optimism indicate a strong belief in the potential of UI profiles to enhance modular and interoperable risk management processes.

### 6.6 Reflection on neutral responses

Across all answers, there has been a substantial number of neutral responses in the Likert Scale. On one hand, this could indicate limited familiarity with the concept of machine-readable UI Profiles. On the other hand, it could show that the participants hesitated to answer before seeing a validation of the evaluated user interfaces. Future studies should identify the actual reason for the neutral responses by performing interviews or adding text fields to gather more feedback.

### 6.7 Limitations and response bias

The participants of the survey had heard of the concept of UI Profiles and had varying knowledge of the topic prior to the workshop. Those with experience in UI Profiles might be biased to give more positive or negative ratings. Additionally, the small sample size limits the power of the statistical analysis and the generalizability of the findings. While we performed a One-Sample Wilcoxon Test to see whether each statement significantly differed from the scale’s neutral point, these exploratory results must be interpreted with caution. With a larger sample, the statistical significance and effect sizes could be more robustly estimated. We acknowledge this as a major limitation of our current study.

The questionnaire was developed by drawing on established risk-management categories derived from ISO 14971 and usability categories from IEC 62366-1 and DIN 9241 standards, combined with preliminary expert reviews from associated researcher. However, the questionnaire has not undergone formal validation. Future work should apply deeper validation methods to strengthen the reliability and validity of our survey. Lastly, the absence of formative real-world evaluations of user interfaces developed based on UI Profiles is a limitation of this work and should be performed in future work.

### 6.8 Ethical considerations

This study did not involve patient data or patient contact. All participating medical device manufacturers were informed about the scope and the purpose of the study. All responses were treated confidentially. No personal data (name, age, gender) has been recorded. No formal ethics committee approval was needed.

## 7 Conclusion

The integration of User Interface Profiles into the existing SDC architecture and standardization presents a pivotal advancement in medical device interoperability within the clinic and the OR. Our findings, supported by comprehensive feedback from medical device manufacturers through a questionnaire, highlight the critical need and benefits of standardized UI Profiles.

Statements regarding the “development and testing phase” received particularly positive and statistically significant ratings, suggesting that medical device manufacturers find UI Profiles especially valuable for guiding design and validation processes. By contrast, most other statements showed weaker (*p* < 0.10) or no statistical significance—outcomes likely influenced by a small pool of respondents rather than an absence of meaningful effects. Consequently, while these results underscore the potential benefits of UI Profiles, they also highlight the need for broader participation to solidify the statistical power and generalizability of the findings.

A majority of respondents agreed that current SDC standards lack effective methods for sharing risk-related requirements for safe device usage, highlighting the existing gap that the UI Profile aims to fill. Despite a significant reluctance to share sensitive risk management files with third parties, manufacturers still wish to limit how other SDC participants use their devices, indicating a clear need for UI Profiles.

Responses indicated high agreement that UI Profiles can serve as input for the design phase, supporting consistency and standardization. Additionally, feedback showed that UI Profiles has the potential to support risk management through the identification and mitigation of human-induced risks. However, the high percentage of neutrality (37.5%–62.5%) in the risk analysis part suggests a need for concrete and practical examples to demonstrate the benefits of UI Profiles. The participants also had reservations regarding a potential design process speed-up and the question of whether fewer formative tests would be necessary.

From a clinical standpoint, ensuring a consistent and risk-aware user interface across multiple medical devices can significantly enhance patient safety by reducing the likelihood of use errors, especially in high-stress OR environments. Standardized UI Profiles have the potential to minimize confusion arising from inconsistent labeling or UI design, and thus decrease human error rates. Furthermore, designing, verifying, and using GUI elements in line with these standardized profiles can foster the development of user-friendly, device-specific GUIs, improve efficiency, and ultimately improve patient outcomes.

By embedding such requirements into the development lifecycle, organizations can more confidently navigate regulatory pathways, demonstrating alignment with recognized international standards and focusing on patient-centered, safe device interoperability.

While our results show promise, the real-world validation of UI Profiles remains to be confirmed. We have yet to demonstrate the direct clinical impact of UI Profiles. A validation study, including user testing with actual medical personnel in a hospital environment, is currently underway and will provide empirical data on user performance, error rates, and efficiency.

By encapsulating design, risk management, and usability engineering aspects into machine-readable UI Profiles, this approach has the potential to significantly enhance the safety and usability of medical device interactions in open networks. It helps to fulfill regulatory requirements stated by the FDA, Notified Bodies for Medical Devices in Europe, and medical device manufacturers (43). This work addresses a critical gap in current interoperability standards by providing a standardized method for communicating UI requirements across medical devices, ensuring that interfaces are consistent and optimized for user needs and safety requirements.

In actual clinical applications, the use of UI Profiles prior to operation could enable device-to-device tests and provide testable and objective criteria to prevent resource conflicts, such as not having enough or suitable input, and output controls/devices (22), confirmations of critical functions prior to releasing (C-Ray, HF power) or to fulfill requirements by particular device standards (e.g., DIN EN 60601-2-2). Without those or similar UI Profiles, providing “real” interoperability becomes a challenge, since exchanging risk management and usability files are necessary to develop an MDR and FDA-compliant, safe, and usable solution.

The proposal of a concrete schema for UI Profiles marks a significant step toward achieving automated UI generation processes, supporting future dynamic creation of user interfaces that fulfill the specific requirements of various medical devices, potentially revolutionizing the way interfaces are designed and implemented in networked operating rooms.

As the UI Profile language evolves, it will be crucial to continue refining the XML-Schema to remain adaptable to the ever-changing landscape of medical technology and user needs. The collaborative efforts of medical device manufacturers, healthcare professionals, and regulatory bodies will be key in advancing this initiative, aiming for a future where medical devices not only communicate seamlessly but also contribute to safer and more effective medical device control and, therefore, better patient care.

In conclusion, the introduction of UI Profiles into the SDC architecture represents an advancement in open, interoperable, and user-centered medical device control systems. Using a standardized approach to define UI requirements, this initiative paves the way for fulfilling SDC standard requirements, mitigating risks already at the design phase, and creating safe, effective, efficient, and intuitive medical device interfaces.

## Data Availability

The original contributions presented in the study are included in the article/Supplementary Material, further inquiries can be directed to the corresponding author.
