# Supplementary material for: Integrating machine-readable user interface requirements into open networked operating rooms
Source: Front Digit Health. 2025 Jul 21;7:1520584. doi: 10.3389/fdgth.2025.1520584 (PMC12319011; doi:10.3389/fdgth.2025.1520584)
Supplement: Supplementary file 1 [file Datasheet1.pdf]

Statement	p-value	P < 0.05?
1. We currently do not see a reasonable way within the SDC standard to share HMI requirements.	0,0781	Weak Evidence
2. We recommend creating device-specific HMI requirements to support modular risk management.	0,1562	-
3. We do not want to provide our risk management file for potential unknown network participants	0,0156	Statistical Significant
4. We want to define and limit how network participants (consumer) use our device.	0,0781	Weak Evidence
5. UI Profiles can serve as input for the design phase by providing HMI requirements	0,0156	Statistical Significant
6. UI Profiles can specify requirements for human-machine interactions.	0,0156	Statistical Significant
7. UI Profiles support consistency and standardization.	0,0078	Statistical Significant
8. UI Profiles would speed up the design process for safe and usable interfaces.	N/A	-
9. UI Profiles would reduce the number of formative usability tests.	N/A	-
10. UI Profiles ensure that users are adequately and correctly informed during device operation	0,0625	Weak Evidence
11. UI Profiles support safe, effective, efficient, and learnable medical device control.	0,0312	Statistical Significant
12. UI Profiles could preemptively identify potential human-induced risks associated with device operation	0,3125	-
13. UI Profiles could meticulously and systematically analyze and decrease UI deficiencies	N/A	-
14. UI Profiles could reduce the frequency of use errors	0,1875	-
15. UI Profiles could reduce cognitive load by providing standardized and consistent UI	0,0625	Weak Evidence
16. UI Profiles could support compliance with SDC Standard Requirements	0,0625	Weak Evidence
17. UI Profiles support systematic and effective risk management	N/A	-
18. We can manage the development of UI Profiles for our devices with in-house resources	0,0156	Statistical Significant
19. We would prefer getting external expertise to develop UI Profiles	N/A	-
20. Sharing process-related risks using a UI Profile is a viable option for us	0,0547	Weak Evidence
